# Supplementary material for: Resolving coral temperature vulnerability through heat and cold bleaching thresholds
Source: Commun Biol. 2025 Dec 20;9:61. doi: 10.1038/s42003-025-09329-5 (PMC12804958; doi:10.1038/s42003-025-09329-5)
Supplement: Supplementary file 2 — Supplementary Information [file 42003_2025_9329_MOESM2_ESM.pdf]

Supplementary Material

Resolving Coral Temperature Vulnerability through Heat and Cold Bleaching Thresholds

Yusuf C. El-Khaled[1*], Francisca C. García[1], Neus Garcias-Bonet[1], Matteo Monti[1], Erika P. Santoro[1], Matilde Marques[2,3], Natalie Dunn[1], Tina Keller-Costa[2,3], Christian R. Voolstra[4], Raquel S. Peixoto[1,5]

*corresponding author: Yusuf Christian El-Khaled, yusuf.khaled@kaust.edu.sa

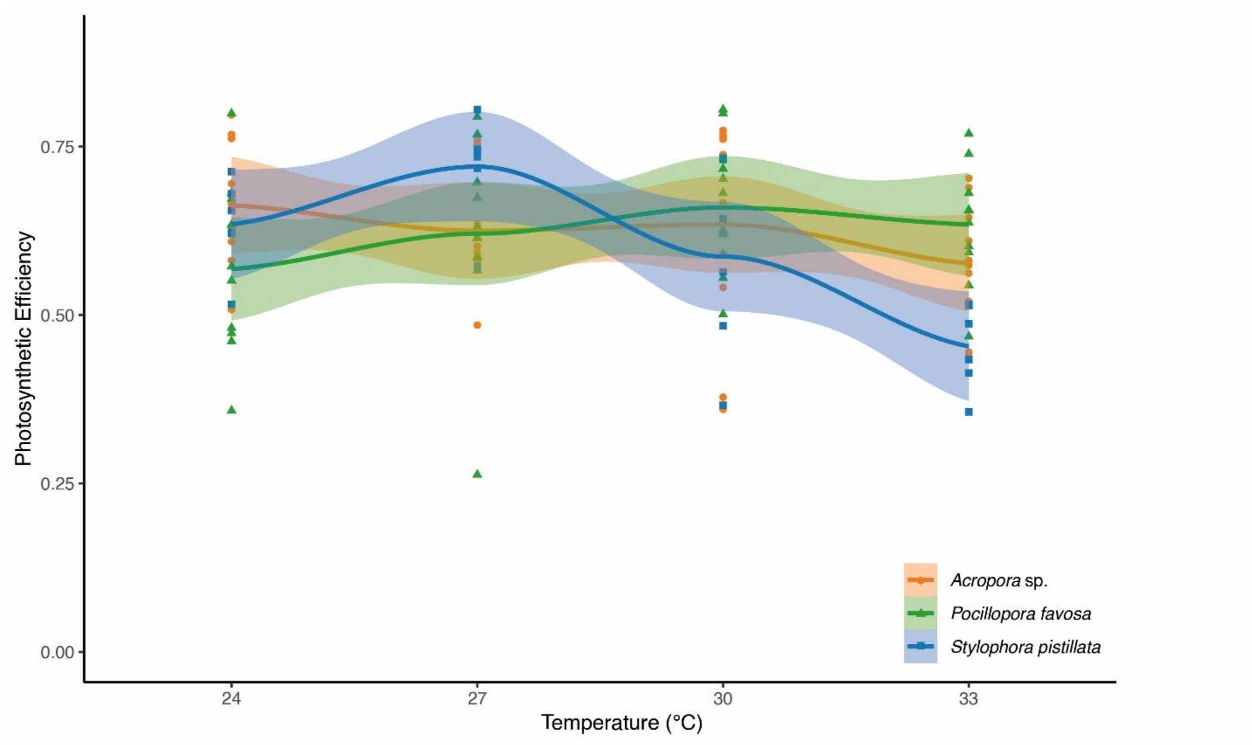

Supplementary Figure 1: Photosynthetic efficiency measurements of all investigated species in winter 2024 at 24-33°C. Photosynthetic efficiency (F_v/F_m) of *Acropora* sp. (orange, n = 10), *Pocillopora favosa* (green, n = 10) and *Stylophora pistillata* (blue, n = 6) measured in classical CBASS assay conducted in winter (February) 2024 using 24°C, 27°C, 30°C and 33°C as target temperatures, with shaded areas representing 95% confidence intervals of the fitted LOESS curves. Chosen temperatures did not alter the photosynthetic efficiency of the hard coral species below 50 % to calculate the corresponding thermal tolerance thresholds (ED50).

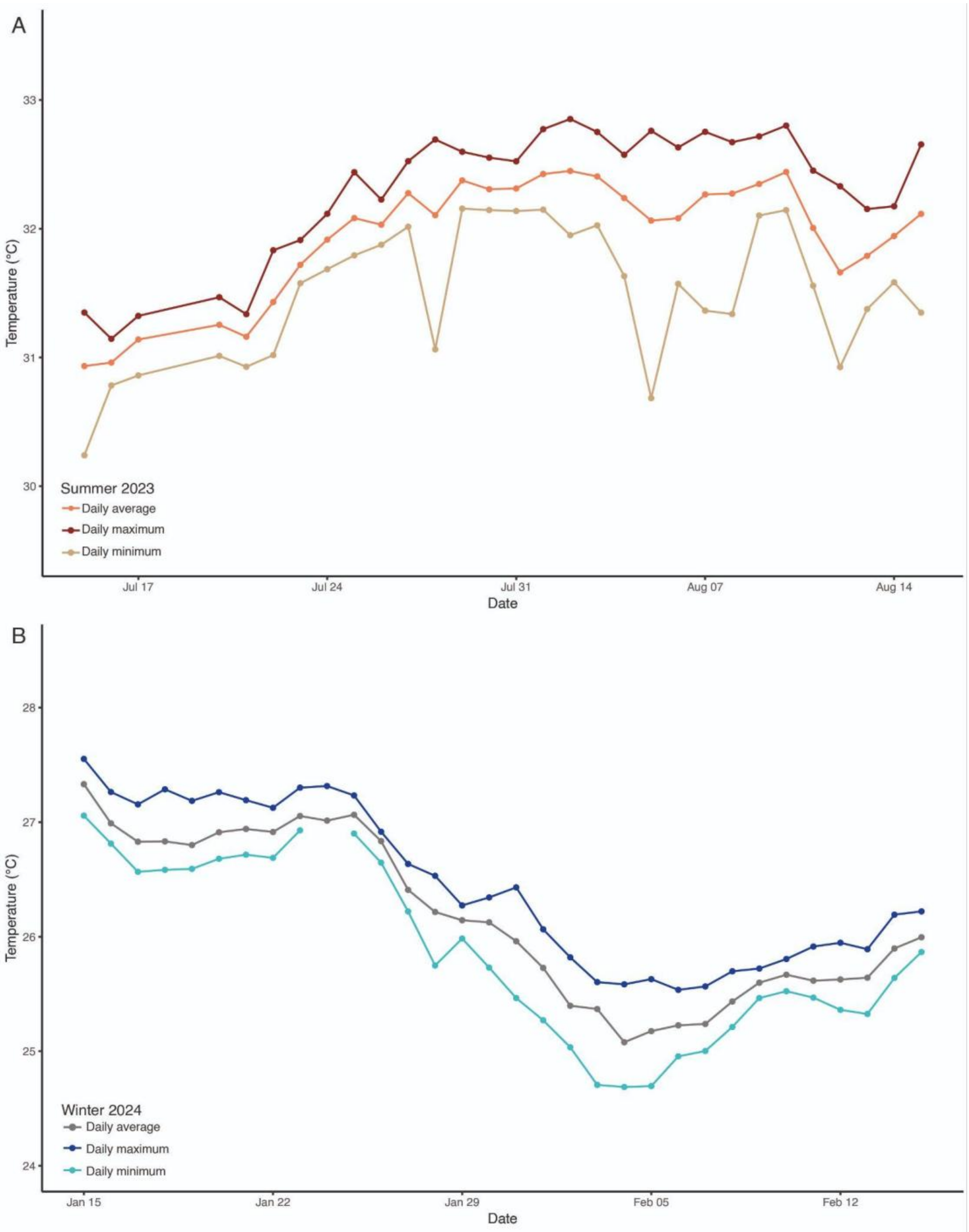

Supplementary Figure 2: Daily average, maximum and minimum *in-situ* temperatures in summer 2023 and winter 2024. Temperatures were measured in the Coral Probiotic Village (CPV), Al Fahal reef, Central Red Sea, before and during the experimental periods in summer 2023 **(A)** and winter 2024 **(B)**. We refer to (Garcias-Bonet et al., (2025) for further details (logger details, replication, etc.).

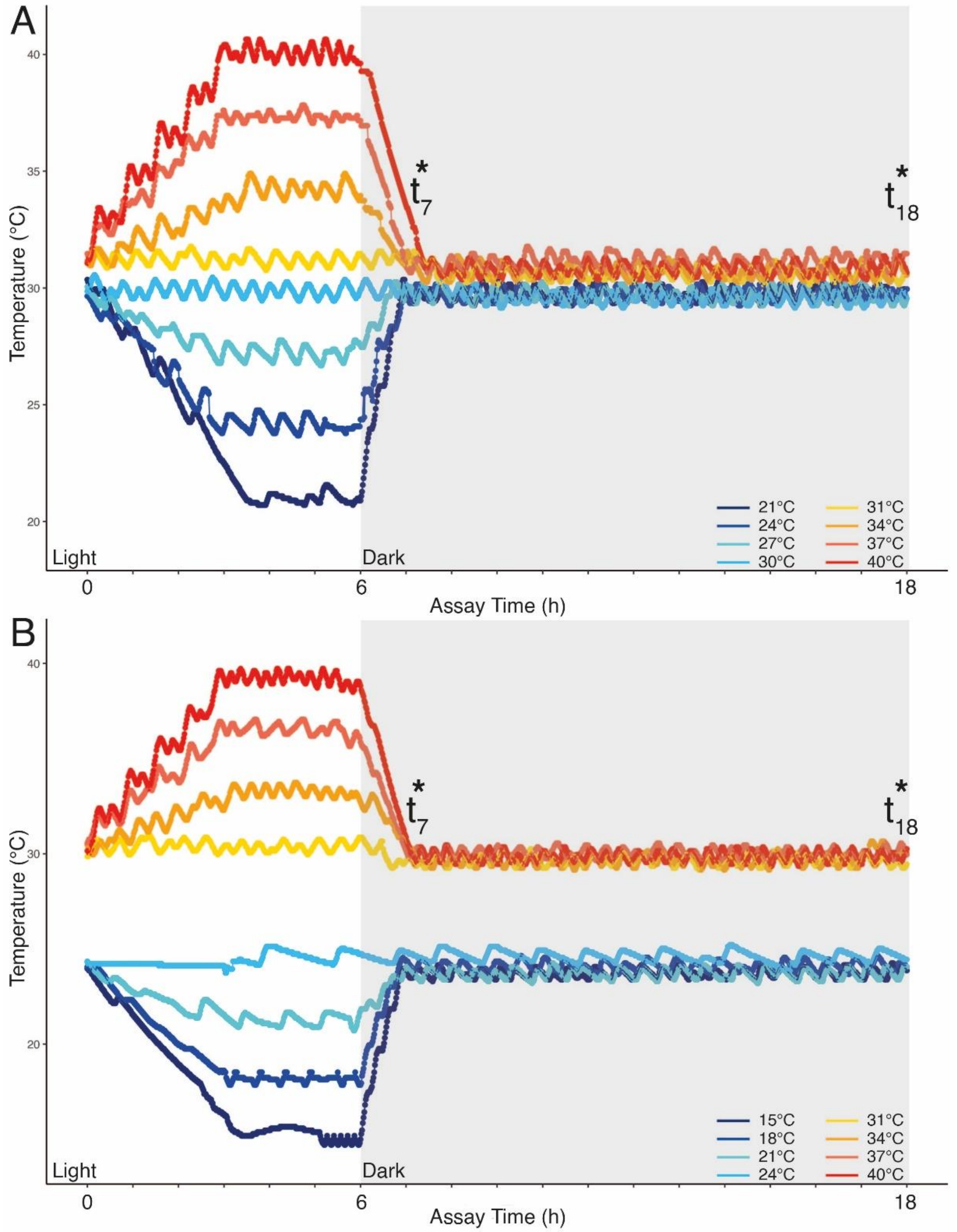

Supplementary Figure 3: Temperature profiles for 18-hour heat and cold acute temperature stress assays. Reported assay profiles were conducted in summer 2023 **(A)** and winter 2024 **(B)**. Start and end times for the assays are indicated below the x-axis; asterisks indicate the time point of dark-adapted photosynthetic efficiency measurements immediately after the stress phase (t_7) and after an 11-hour recovery period (t_{18}), temperatures were measured at a 1-minute interval using a HOBO Pendant Onset logger (raw data in online repository (El-Khaled et al., 2025)).

Supplementary Table 1: Results of Spearman rank correlation for heat (red) and cold (blue) ED50 thresholds for the three most dominant bacterial families (*Endozoicomonaceae*, *Rhodobacteraceae*, *Simkaniaceae*) for all three coral species of the present study during summer (heat ED50; n = 9 for *Acropora* sp. and *P. favosa*, and n = 7 for *S. pistillata*) and winter (cold ED50; n = 9 for *Acropora* sp., n = 10 for *P. favosa*, and n = 6 for *S. pistillata*)), with rho values indicating the strength and direction of the investigated relationships and *p*-values indicating statistical significance with $p < 0.05$ (values in bold). Asterisks indicate values close to statistical significance. NA indicating insufficient replication.

	Endozoicomonadaceae		*Rhodobacteraceae*		*Simkaniaceae*	
	rho	*p* value	rho	*p* value	rho	*p* value
ED50 x summer & winter x all species	0.2892	**0.0462**	0.321	**0.0261**	0.2727	0.0608*
ED50 x summer x all species	0.2949	0.1528	0.4177	**0.0378**	NA	NA
ED50 x summer x *Acropora* sp.	-0.709	**0.0216**	0.5272	0.1173	NA	NA
ED50 x summer x *Pocillopora favosa*	0.3333	0.4198	0.4524	0.2604	NA	NA
ED50 x summer x *Stylophora pistillata*	0.0357	0.9394	NA	NA	NA	NA
ED50 x summer & winter x all species	0.3594	**0.0179**	0.2977	0.0526*	0.2785	0.0706*
ED50 x winter x all species	0.3038	0.1838	0.3068	0.1883	NA	NA
ED50 x winter x *Acropora* sp.	0.2	0.704	-0.3714	0.4685	NA	NA
ED50 x winter x *Pocillopora favosa*	0.5281	0.1827	-0.2143	0.6103	NA	NA
ED50 x winter x *Stylophora pistillata*	0.4286	0.3965	0.7714	0.0724*	NA	NA

Supplementary Table 2: Comparisons of calculated ED50 from classical and cold CBASS assays in summer and winter for *Acropora* sp., *Pocillopora favosa*, and *Stylophora pistillata* presenting Dunn's multiple comparison post-hoc test. Given values are adjusted *p*-values with the Holm method (see method section for further details). In case 'n/a' is reported, Kruskal Wallis test did not show any significant results. Significant values with $p < 0.05$ are given in bold.

Comparison	Classical CBASS - Summer	Cold CBASS - Summer	Classical CBASS- Winter	Cold CBASS-Winter
Acropora sp. - *P. favosa*	**0.026**	0.099	n/a	0.15
Acropora sp. - *S. pistillata*	0.395	0.59	n/a	**0.012**
P. favosa - *S. pistillata*	0.257	0.076	n/a	0.147

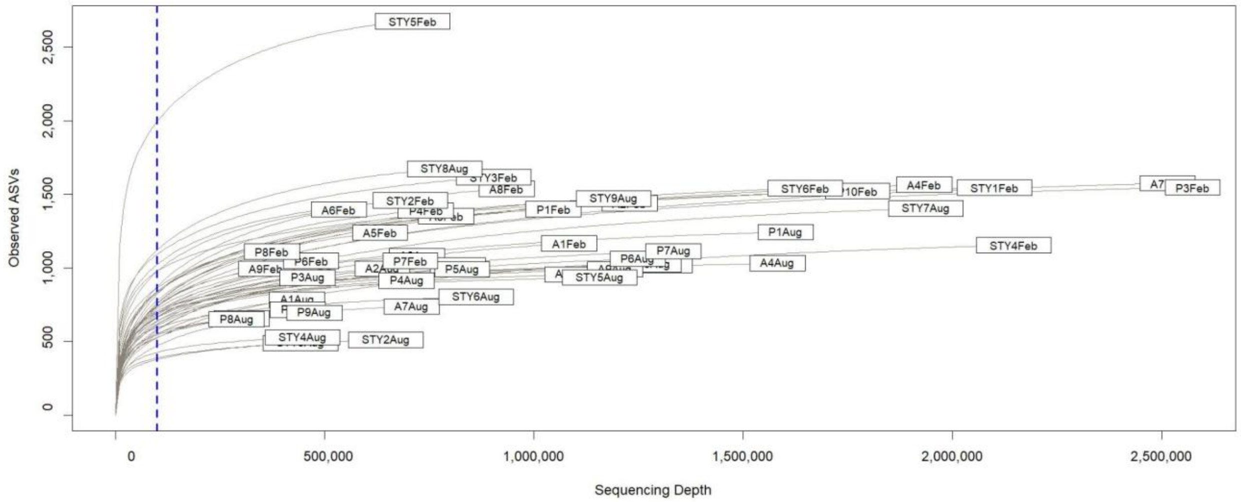

Supplementary Figure 4: Rarefaction curve of microbiome data used for analysis. Rarefaction was applied at 100,000 reads (blue dotted line) for all specimens of the three targeted coral species' microbiome data (*Acropora* sp. = 'A'; *Pocillopora favosa* = 'P"'; and *Stylophora pistillata* = 'STY', respectively) for both summer 2023 ('Aug') and winter 2024 ('Feb'). Replication is n = 10 and n = 9 for *Acropora* sp. in summer and winter, respectively, n = 9 and n = 8 for *P. favosa* in summer and winter, respectively, and n = 8 and n = 6 for *S. pistillata* in summer and winter, respectively.

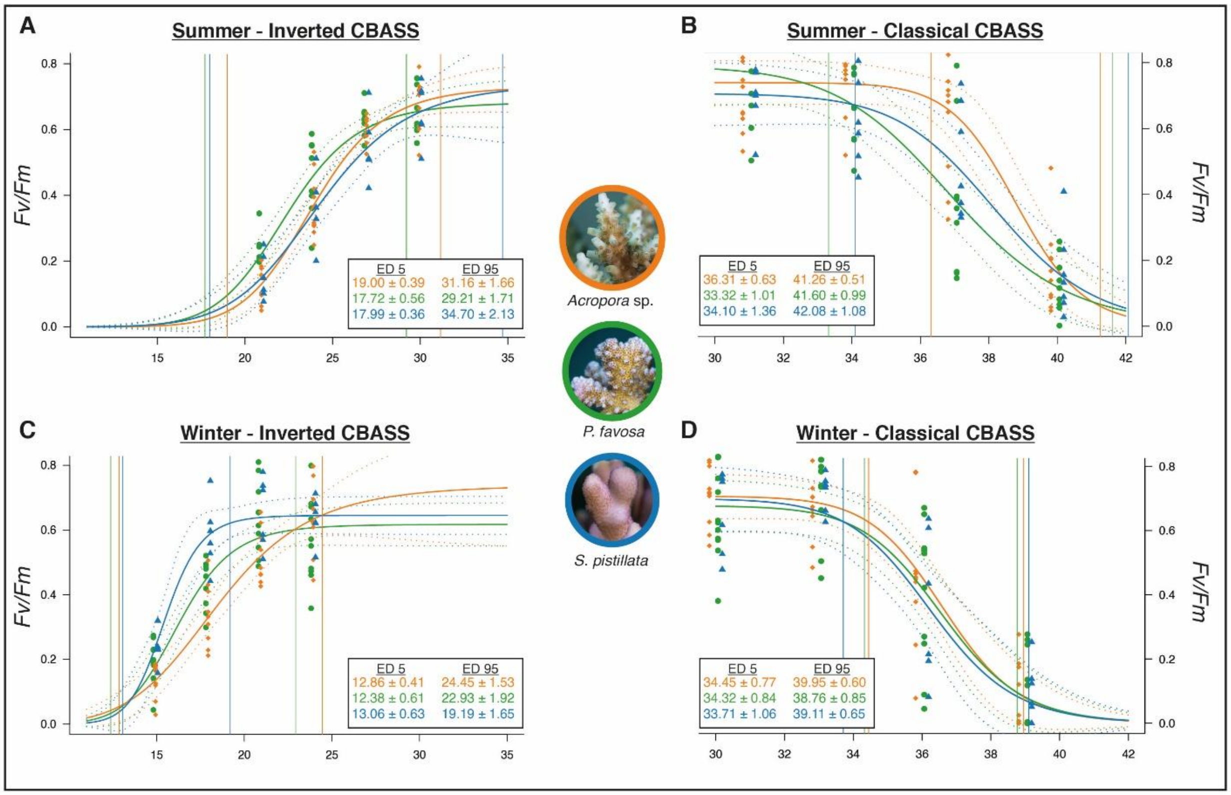

Supplementary Figure 5: Calculated effective dose 5 (ED5) and effective dose 95 (ED95) changes in photosynthetic efficiency (*Fv/Fm*). ED5 and ED95 changes are displayed with cold **(A, C)** and heat **(B, D)** assay temperature profiles for each CBASS experiment performed in summer **(A, B)** and winter **(C, D)** using *Acropora* sp. (orange, rectangles, n = 8 in A, n = 9 in B and C; n = 10 in D), *Pocillopora favosa* (green, dots, n = 8 in A; n = 9 in B; n = 10 in C and D) and *Stylophora pistillata* (blue, triangles, n = 6 in A, C and D, n = 7 in B). Dots, triangles and squares, respectively, represent the measured photosynthetic efficiency (*Fv/Fm*) at each experimental temperature for each CBASS assay and species. Lines reflect the log-logistic model fitted to each experiment (see methods) with dotted lines indicating the 95% confidence intervals of each log-logistic model. Color-coded temperature values show the ED5 and ED95 of each species in °C ± standard error. Representative pictures of the coral species taken by Matteo Monti.

Supplementary Table 3: Decline width (*DW*, in °C) expressed as *DW* = ED95 – ED5 (see Supplementary Fig. S5) as an approximation to describe the shape of the fitted F_v/F_m response curves (wide or narrow) as shown in Fig. 2 and Supplementary Figure S5.

Species	Heat CBASS - Summer	Cold CBASS - Summer	Heat CBASS - Winter	Cold CBASS - Winter
Acropora sp.	4.95 ± 0.92	12.16 ± 2.11	4.50 ± 1.11	11.59 ± 1.57
Pocillopora facosa	8.28 ± 1.99	11.50 ± 2.28	4.44 ± 1.57	10.53 ± 2.44
Stylophora pistillata	7.98 ± 2.52	16.71 ± 2.59	5.40 ± 1.50	6.13 ± 2.50

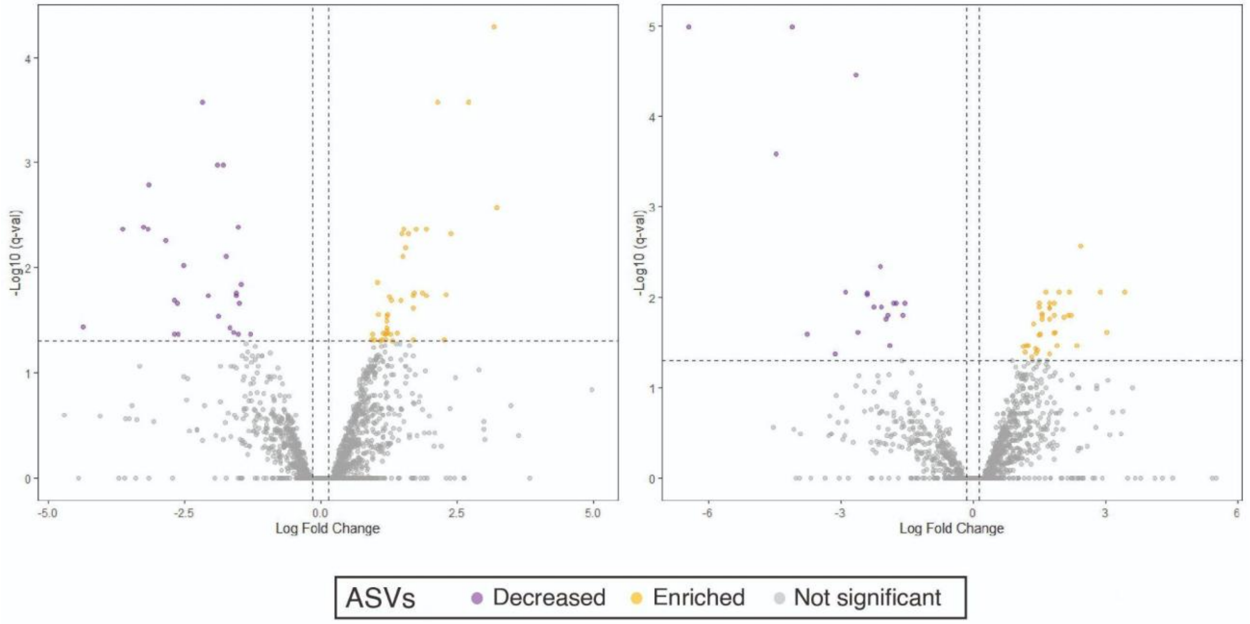

Supplementary Figure 6: Volcano plots displaying differentially abundant ASVs (dots) identified in the ANCOM-BC2 analysis. Plots display *Acropora* sp. (left; n = 69 differentially abundant ASVs in summer compared to winter) and *Pocillopora favosa* (right; n = 49 differentially abundant ASVs in summer compared to winter), with n = 10 and n = 9, respectively for *Acropora* sp. in summer and winter, respectively; and n = 9 and n = 8 for *P. favosa* in summer and winter, respectively. The log fold change (*X*-axis) and the *p*-adj. (*Y*-axis) value for each ASV is represented. Yellow dots indicate enriched ASVs and purple dots indicate decreased ASVs with a *q* value (adjusted *p* value) < 0.05. ASVs that are not significantly different in abundance between seasons (summer vs. winter) are colored gray. Note: Different scales for y-axes; no significant changes in ASVs between summer and winter were observed for *Stylophora pistillata*, hence it is missing in this figure.

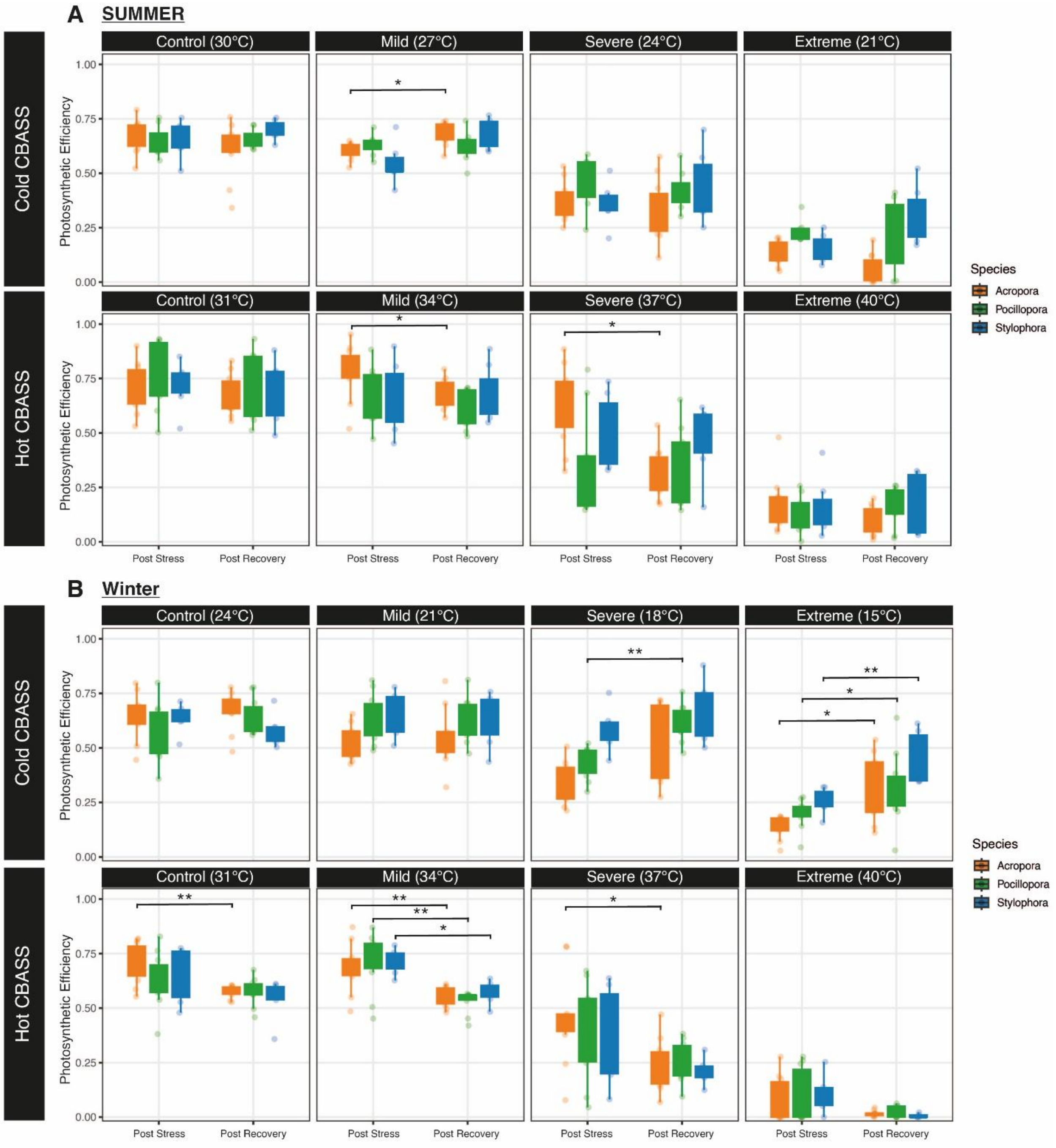

Supplementary Figure 7: Photosynthetic efficiency (F_v/F_m) measured during all CBASS assays temperatures. Photosynthetic efficiency was measured during summer **(A)** and winter **(B)**, and with measurements performed immediately after the stress phase (t7) and an 11-hour recovery phase (t18) for *Acropora* sp. (orange, n = 8 in cold CBASS in summer, n = 9 in hot CBASS in summer and cold CBASS in summer; n = 10 in hot CBASS in winter), *Pocillopora favosa* (green, n = 8 in cold CBASS in summer; n = 9 in hot CBASS in summer; n = 10 in both hot and cold CBASS in winter), and *Stylophora pistillata* (blue, , n = 6 in cold CBASS in summer and winter, and hot CBASS in winter, n = 7 in hot CBASS in summer). Boxplots show photosynthetic efficiency (*Fv/Fm*) of the three coral species, with boxes

representing the interquartile range (IQR) with the median line; whiskers extend to 1.5× IQR, and jittered points denote individual replicates. Significant differences based on paired t-test or Wilcoxon signed-rank test are displayed using asterisks, with * for $p < 0.05$, ** for $p < 0.01$, and *** for $p < 0.001$.

Supplementary References

El-Khaled, Y. C., Garcia, F. C., Garcias-Bonet, N., Monti, M., Santoro, E. P., Dunn, N., Marques, M., Keller-Costa, T., Voolstra, C. R., & Peixoto, R. S. (2025). Resolving Heat but also Cold Bleaching Thresholds Predicts Coral Temperature Vulnerability - Raw data for analysis, supplementary data, and R-Code. In *Resolving Heat but also Cold Bleaching Thresholds Predicts Coral Temperature Vulnerability*. https://doi.org/10.5281/zenodo.15124619. https://doi.org/10.5281/zenodo.15124619

Garcias-Bonet, N., Villela, H., García, F. C., Duarte, G. A. S., Delgadillo-Ordoñez, N., Raimundo, I., El-Khaled, Y. C., Santoro, E. P., Bennett-Smith, M., Nieuwenhuis, B. O., Curdia, J., Zgliczynski, B., Edwards, C., Sandin, S., Osman, E. O., Sicat, R., Przybysz, A., Rosado, A. S., Jones, B. H., … Peixoto, R. S. (2025). The Coral Probiotics Village: An Underwater Laboratory to Tackle the Coral Reefs Crisis. *Ecology and Evolution*, *15*(7). https://doi.org/10.1002/ece3.71558
