# Supplementary material for: Resolving coral temperature vulnerability through heat and cold bleaching thresholds
Source: Commun Biol. 2025 Dec 20;9:61. doi: 10.1038/s42003-025-09329-5 (PMC12804958; doi:10.1038/s42003-025-09329-5)
Supplement: Supplementary file 3 — Description of Additional Supplementary Files [file 42003_2025_9329_MOESM3_ESM.pdf]

**Description of Additional Supplementary Files**

**File name:** Supplementary_Data_1.xlsx

**Description:** Source data behind Figure 1

**File name:** Supplementary_Data_2.xlsx

**Description:** Additional information to statistical analyses
