# Supplementary material for: Resolving coral temperature vulnerability through heat and cold bleaching thresholds
Source: Commun Biol. 2025 Dec 20;9:61. doi: 10.1038/s42003-025-09329-5 (PMC12804958; doi:10.1038/s42003-025-09329-5)
Supplement: Supplementary file 6 — Reporting Summary [file 42003_2025_9329_MOESM6_ESM.pdf]

nature portfolio

Corresponding author(s): Yusuf C. El-Khaled, Raquel S. Peixoto

Last updated by author(s): Nov 11, 2025

# Reporting Summary

Nature Portfolio wishes to improve the reproducibility of the work that we publish. This form provides structure for consistency and transparency in reporting. For further information on Nature Portfolio policies, see our Editorial Policies and the Editorial Policy Checklist.

## Statistics

For all statistical analyses, confirm that the following items are present in the figure legend, table legend, main text, or Methods section.

| n/a | Confirmed | |
|---|---|---|
| ☐ | ☒ | The exact sample size (*n*) for each experimental group/condition, given as a discrete number and unit of measurement |
| ☐ | ☒ | A statement on whether measurements were taken from distinct samples or whether the same sample was measured repeatedly |
| ☐ | ☒ | The statistical test(s) used AND whether they are one- or two-sided<br>*Only common tests should be described solely by name; describe more complex techniques in the Methods section.* |
| ☐ | ☒ | A description of all covariates tested |
| ☐ | ☒ | A description of any assumptions or corrections, such as tests of normality and adjustment for multiple comparisons |
| ☐ | ☒ | A full description of the statistical parameters including central tendency (e.g. means) or other basic estimates (e.g. regression coefficient) AND variation (e.g. standard deviation) or associated estimates of uncertainty (e.g. confidence intervals) |
| ☐ | ☒ | For null hypothesis testing, the test statistic (e.g. *F*, *t*, *r*) with confidence intervals, effect sizes, degrees of freedom and *P* value noted<br>*Give P values as exact values whenever suitable.* |
| ☒ | ☐ | For Bayesian analysis, information on the choice of priors and Markov chain Monte Carlo settings |
| ☒ | ☐ | For hierarchical and complex designs, identification of the appropriate level for tests and full reporting of outcomes |
| ☒ | ☐ | Estimates of effect sizes (e.g. Cohen's *d*, Pearson's *r*), indicating how they were calculated |

*Our web collection on statistics for biologists contains articles on many of the points above.*

## Software and code

Policy information about availability of computer code

| | |
|---|---|
| Data collection | Description of how data were collected is part of the method section. |
| Data analysis | A reproducible description of how data were analysed is part of the method section. We have also uploaded all R codes used in the manuscript to a repository that will be publicly accessible upon publication. |

For manuscripts utilizing custom algorithms or software that are central to the research but not yet described in published literature, software must be made available to editors and reviewers. We strongly encourage code deposition in a community repository (e.g. GitHub). See the Nature Portfolio guidelines for submitting code & software for further information.

## Data

Policy information about availability of data

All manuscripts must include a data availability statement. This statement should provide the following information, where applicable:

- Accession codes, unique identifiers, or web links for publicly available datasets
- A description of any restrictions on data availability
- For clinical datasets or third party data, please ensure that the statement adheres to our policy

All raw sequence reads were deposited in the ENA under the study accession number PRJEB88613. The R scripts, raw data, results of differentially abundant ASVs for each species at the different seasons as well as the results of the ANCOM-BC2 analyses are available online 131.

## Research involving human participants, their data, or biological material

Policy information about studies with human participants or human data. See also policy information about sex, gender (identity/presentation), and sexual orientation and race, ethnicity and racism.

| | |
|---|---|
| Reporting on sex and gender | NA |
| Reporting on race, ethnicity, or other socially relevant groupings | NA |
| Population characteristics | NA |
| Recruitment | NA |
| Ethics oversight | NA |

Note that full information on the approval of the study protocol must also be provided in the manuscript.

# Field-specific reporting

Please select the one below that is the best fit for your research. If you are not sure, read the appropriate sections before making your selection.

☐ Life sciences ☐ Behavioural & social sciences ☒ Ecological, evolutionary & environmental sciences

For a reference copy of the document with all sections, see nature.com/documents/nr-reporting-summary-flat.pdf

# Ecological, evolutionary & environmental sciences study design

All studies must disclose on these points even when the disclosure is negative.

| | |
|---|---|
| Study description | Building upon the widely used ED50 metric for standardized heat tolerance, we introduce a new metric, cold ED50, to quantify cold bleaching thresholds. By comparing cold and heat ED50s, we define the temperature variability range of coral species. To achieve this, we used 'Coral Bleaching Automated Stress System' ('CBASS') assays to assess heat and cold temperature tolerance across three Red Sea scleractinian corals. |
| Research sample | Three Red Sea scleractinian corals (Acropora sp., Pocillopora verrucosa, Stylophora pistillata) and their associated microbiome assessed via DNA extraction and sequencing using 16S primer |
| Sampling strategy | Coral fragments for both heat and cold CBASS assays were collected from the same set of tagged coral colonies (i.e., distinct but clonal fragments for each assay). For cold CBASS assays, coral fragments were collected on August 15th, 2023, and on February 14th, 2024; coral fragments for heat CBASS were collected on August 20th, 2023, and on February 18th, 2024, respectively |
| Data collection | 1) Dark-acclimated photosynthetic efficiencies, i.e., the maximum photosystem (PS) II quantum yield (Fv/Fm), were measured by the research team.<br>2) Additionally, one coral fragment of each genotype was collected per sampling season that was used for microbial profiling. |
| Timing and spatial scale | For cold CBASS assays, coral fragments were collected on August 15th, 2023, and on February 14th, 2024; coral fragments for heat CBASS were collected on August 20th, 2023, and on February 18th, 2024, respectively.<br>We started the experiments at the wet lab facility of the Coastal and Marine Resources (CMOR) Core Lab at the King Abdullah University of Science and Technology (KAUST) using the Coral Bleaching Automated Stress System (CBASS) less than three hours after sampling, using water collected from the reef site. During these three hours, coral fragments were kept under ambient temperatures aiming to bypass any possible acclimation and to avoid adjustments to possible tank conditions aiming to have close to in-situ conditions. |
| Data exclusions | No data were excluded. |
| Reproducibility | We are using an established approach, the Coral Bleaching Automated Stress System (CBASS) for which we modified the temperature settings. We provide a full list of the experimental settings to ensure reproducibility. Further, all codes and raw sequences are provided. |
| Randomization | Coral genotypes were randomly selected from the same sampling area. |
| Blinding | Not relevant for the present study. |

Did the study involve field work? ☐ Yes ☐ No

## Field work, collection and transport

| | |
|---|---|
| Field conditions | We report in situ temperature data in the manuscript of the times when the corals have been collected (see Supplementary Figure 2). As such, we used the mean minimum temperature of July 2023 (here 30.5°C) as a control. In February, we similarly choose the mean minimum temperature of January 2024 (24°C, respectively; Supplementary Fig. S2). |
| Location | This study has been performed in the Coral Probiotic Village in the Central Red Sea (see Garcias-Bonet et al. 2025 for further information). |
| Access & import/export | Sailing permits were issued to the Coastal and Marine Operations Labs associated with the King Abdullah University of Science and Technology by the Saudi Government. |
| Disturbance | No disturbance. |

# Reporting for specific materials, systems and methods

We require information from authors about some types of materials, experimental systems and methods used in many studies. Here, indicate whether each material, system or method listed is relevant to your study. If you are not sure if a list item applies to your research, read the appropriate section before selecting a response.

### Materials & experimental systems

| n/a | Involved in the study | |
|---|---|---|
| ☒ | ☐ | Antibodies |
| ☒ | ☐ | Eukaryotic cell lines |
| ☒ | ☐ | Palaeontology and archaeology |
| ☐ | ☒ | Animals and other organisms |
| ☒ | ☐ | Clinical data |
| ☒ | ☐ | Dual use research of concern |
| ☒ | ☐ | Plants |

### Methods

| n/a | Involved in the study | |
|---|---|---|
| ☒ | ☐ | ChIP-seq |
| ☒ | ☐ | Flow cytometry |
| ☒ | ☐ | MRI-based neuroimaging |

## Animals and other research organisms

Policy information about studies involving animals; ARRIVE guidelines recommended for reporting animal research, and Sex and Gender in Research

| | |
|---|---|
| Laboratory animals | The study did not involve laboratory animals. |
| Wild animals | Three Red Sea scleractinian corals (Acropora sp., Pocillopora verrucosa, Stylophora pistillata) and their associated microbiome. |
| Reporting on sex | This information has not been collected. |
| Field-collected samples | After collection, all coral fragments were immediately transferred to temporary aquaria filled with freshly on-site sampled reef water on the boat and kept at ambient light and temperatures. An extra fragment of each genotype was sampled for microbial analysis (see sections below). We started the experiments at the wet lab facility of the Coastal and Marine Resources (CMOR) Core Lab at the King Abdullah University of Science and Technology (KAUST) using the Coral Bleaching Automated Stress System (CBASS) less than three hours after sampling, using water collected from the reef site. During these three hours, coral fragments were kept under ambient temperatures aiming to bypass any possible acclimation and to avoid adjustments to possible tank conditions aiming to have close to in-situ conditions. |
| Ethics oversight | This study was conducted under the Institutional Biosafety and Bioethics Committee (IBEC) approval (23IBEC097) following the guidelines of the Kingdom of Saudi Arabia National Committee of Bioethics (KSA NCBE). |

Note that full information on the approval of the study protocol must also be provided in the manuscript.

## Plants

| | |
|---|---|
| Seed stocks | NA |
| Novel plant genotypes | NA |
| Authentication | NA |
